# Effects of static dosimetric leaf gap on MLC‐based small‐beam dose distribution for intensity‐modulated radiosurgery

**DOI:** 10.1120/jacmp.v8i4.2397

**Published:** 2007-10-24

**Authors:** Jeong‐Woo Lee, Kyoung‐Sik Choi, Semie Hong, Yon‐Lae Kim, Jin‐Beom Chung, Doo‐Hyun Lee, Bo‐Young Choe, Hong‐Seok Jang, Tae‐Suk Suh

**Affiliations:** ^1^ Department of Radiation Oncology Konkuk University School of Medicine, Konkuk University Hospital; ^2^ Department of Biomedical Engineering The Catholic University of Korea School of Medicine; ^3^ Department of Radiation Oncology Ajou University School of Medicine, Ajou University Hospital; ^4^ Department of Radiation Oncology The Catholic University of Korea School of Medicine Seoul Republic of Korea

**Keywords:** MLC‐based small beam, dosimetric static leaf gaps

## Abstract

The aim of the present study was to evaluate the effect of various specific dosimetric leaf gaps on the multileaf collimator (MLC)–based small‐beam dose distribution. The dosimetric static leaf gap was determined by comparing the profiles of small MLC‐based beams with those of small collimated fields (square fields of 1, 2, 3, and 4cm). The results showed that an approximately 2‐mm gap was optimal with the Millennium 120‐leaf MLC (Varian Medical Systems, Palo Alto, CA) and a Varian 21EX 6‐MV photon beam. We also investigated how much the leaf gap affects the planning results and the actual dose distribution. A doughnut‐shaped planning target volume (PTV, 6.1 cm^3^) and inner organ at risk (OAR, 0.3 cm^3^) were delineated for delicate intensity‐modulated radiosurgery test planning. The applied leaf gaps were 0, 1, and 2 mm. The measured dose distributions were compared with the dose distribution in the treatment planning system. The maximum dose differences at inside PTV, outside PTV, and inner OAR were, respectively, 22.3%, 20.2%, and 35.2% for the 0‐mm leaf gap; 17.8%, 22.8%, and 30.8% for the 1‐mm leaf gap; and 5.5%, 8.5%, and 6.3% for the 2‐mm leaf gap. In a human head phantom (model 605: CIRS, Norfolk, VA) study, large dose differences of 1.3% – 12.7% were noted for the measurements made using the MLC files generated by the three different leaf gaps. The planned results were similar, and measurements showed a large dose difference associated with the various leaf gaps. These results strongly suggest that plans generated by a commercial inverse planning system commissioned using general collimated field data will probably demonstrate discrepancies between the planned treatments and the measured results.

PACS number: 87.53.Dq

## I. INTRODUCTION

Use of small beams is not restricted merely to radiosurgery; it is also widespread in intensity‐modulated therapy. Small beamlets in intensity‐modulated radiotherapy (IMRT) or radiosurgery (IMRS) are commonly used to optimize the dose distribution. During the optimization of IMRT or IMRS, many small beamlets are generated in complex multiple sequences of fields. Moreover, because the small beamlets used in multileaf collimator (MLC)–based intensity‐modulated fields present complex interactions such as rounded leaf‐end effect,^(1)^ tongue‐and‐groove effect,^(2)^ and lateral disequilibrium,^(3)^ the field of radiation therapy physics requires that considerable attention be paid to planning and dosimetry. Much scientific work has been done on the topic of multileaf collimation and small‐beam dosimetry, but rarely has an attempt been made to connect the two. Generally, the IMRT commissioning process requires specification of a dosimetric leaf gap, which affects the leaf motion calculation (LMC) based on the optimized results^(1)^ and on basic beam data from the jaw‐collimated fields, not MLC fields.

Two concerns arise with collimation using a non‐focused leaf end. First, the penumbra width can be larger than that generated by a focused or divergent edge. Second, the penumbra width might change as a function of the distance of the leaf end from the field midline.^(4)^ The dose fluence generated from the MLC‐based small beam is highly sensitive to inaccuracies in the MLC calibration,^(5)^ and an undesired dose distribution to the planning target volume (PTV) and the organ at risk (OAR) might result.

Although the general dosimetric characteristics of MLC have been described elsewhere,^(^ ^3^ ^–^ ^13^ ^)^ the dosimetric effects of MLC‐based small beams on the actual dose distribution as compared with results from a treatment planning system are unclear—particularly for the small beams used in IMRT and IMRS. In addition, many studies have used stereotactic detectors such as micro‐ionization chambers, silver halide films, and radiochromic films for the commissioning of small‐beam dosimetry and IMRT, but some controversy remains regarding the selection of an optimal detector for small‐beam dosimetry.^(^ ^14^ ^–^ ^18^ ^)^ The dose calculation algorithm in the optimization module of inverse planning systems does not depend on measured commissioning beam data, but rather on Monte Carlo–calculated kernels; however, the LMC depends heavily on dosimetric parameters such as the dosimetric leaf gap, interleaf leakage, and dynamic leaf gap. Because the Varian Eclipse planning system (version 6.5) uses a pencil‐beam convolution (PBC) model that was derived from data gathered using a standard 0.13 cm^3^ ionization chamber, some dosimetric error in the penumbra area could result from the convolution of the spatial response of the ionization chamber with the physical penumbra shape.^(1)^ Although a great deal of discussion has occurred regarding the characteristics of a MLC‐based modulated beam,^(^ ^1^ ^–^ ^13^ ^)^ the main focus of the present study is the leaf‐gap effect, which is believed to be the main source of discrepancy—particularly in a MLC‐based small beam.

Our aim in the present study was to evaluate dose distribution in the MLC‐based small beam using various specific dosimetric leaf gaps.

## II. METHODS AND MATERIALS

### A. Determination of the dosimetric static leaf gap

We used the 6‐MV photon beam from CL 21EX linear accelerator with a 120‐leaf Millennium MLC (Varian Medical Systems, Palo Alto, CA) for our study.

We determined the static dosimetric leaf gap by carrying out profile measurements for MLC‐based small‐beam fields of 1, 2, 3, and 4 cm with four different stereotactic detectors. The stereotactic detectors were a 0.01‐mm ionization chamber with an outer electrode of 2 mm inner diameter (CC01: Wellhofer Dosimetrie, Louvain‐la‐Neuve, Belgium), a stereotactic field detector (SFD: Wellhofer Dosimetrie) with an active area of diameter 0.6+0.003/−0.001 mm, radiographic extended dose range film (EDR2: Eastman Kodak Company, Rochester, NY), and radiochromic film (Gafchromic EBT: International Specialty Products, Wayne, NJ). The film scanner (VXR‐16 Dosimetry Pro: Wellhofer Dosimetrie) was calibrated using step film provided by vendor, and each film was calibrated to the known dose with a CL 21EX linear accelerator before film dosimetry. A water phantom (Blue phantom: Wellhofer Dosimetrie), an electrometer (DOSE1: Wellhofer Dosimetrie), and a source‐to‐surface distance of 95 cm and a depth of 5 cm were used for measurements with the CC01 and SDF.

**Figure 1:**
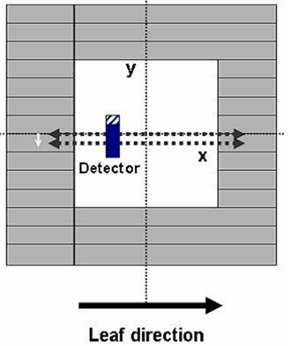
The transversal profiles were measured along the leaf direction. The profiles were measured with an offset of 2.5 mm, positioned in the middle of the leaf to avoid the interleaf gap and to consider the round‐leaf gap effect.

The dosimetric static leaf gap was defined as a 50% radiation MLC field width minus the calibrated field width. The film measurements were acquired using a polystyrene solid phantom (SP33: Wellhofer Dosimetrie). The irradiated films were analyzed using an IMRT film dosimetry system (OmniPro IMRT: Wellhofer Dosimetrie). All profiles were extracted at a position parallel to the leaf direction that was shifted 2.5 mm off‐center to avoid an interleaf gap in the middle of one of the two 5 mm central leaves (Fig. 1). In the present study, the dosimetric leaf gap was set to 2 mm by four different stereotactic detectors through the MLC‐based small‐beam analysis.

### B. Inverse planning with various dosimetric leaf gaps

In previous experiments, MLC‐based small‐beam profiles have shown a definite difference from the profiles of collimated fields because of the rounded leaf‐end effect. We used a commercial treatment planning system (Eclipse version 6.5: Varian Medical Systems) to compare the desired dose distribution produced by the dose‐volume optimizer (DVO) with the actual dose results produced by the LMC using various dosimetric leaf gaps. The Eclipse planning system uses a pencil‐beam convolution algorithm that depends on the user's measured beam data to calculate the dose distribution.^(^ ^19^ ^–^ ^22^ ^)^ During configuration, the Eclipse system requires data only for square collimated fields (usually square fields of 4 – 40 cm), and not MLC‐based fields. The Eclipse DVO engine does not contain the types of errors that arise from kernels derived from inaccurate penumbra measurement. However, the final dose calculation will contain such errors if it is based on commissioning measurements with a standard ionization chamber.^(1)^

To analyze the dependency of the LMC results and the actual dose distribution on the gap values, two additional dummy values of 0 mm and 1 mm for leaf gap were set during the configuration process, and the whole process was repeated, except for image registration. In addition, the PTV was drawn as a doughnut, with very small volume of 0.61 mm, to evaluate the effect of an MLC‐based small beam on the intensity‐modulated fields. Moreover, an extremely small inner OAR, with a volume of 0.3 mm, was set in the middle of the doughnut‐shaped PTV. For this experiment, a commercial stereotactic human head phantom (Model 605: CIRS, Norfolk, VA) was used.

**Figure 2:**
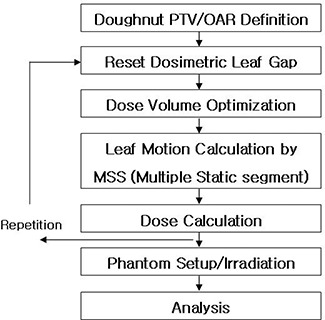
Schematic of the inverse planning and analysis processes with three different dosimetric gaps. The leaf motion calculation considers the dosimetric leaf gaps for the leaf position calculation with respect to the same planning result. PTV=planning target volume; OAR=organ at risk.

**Figure 3:**
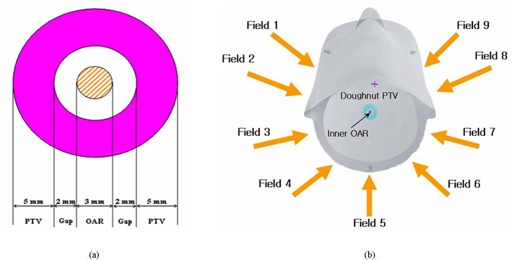
(A) Axial view of a doughnut‐shaped planning target volume (PTV) and inner organ at risk (OAR) in the computed tomography image. (B) Schematic of the applied beam orientation with gantry angles of 310, 280, 250, 220, 180, 140, 110, 80, and 50 degrees. Modulated small fields had the same dimensions: 4.2×3.6 cm (*x*x*y*).

Fig. 2 shows the inverse planning and analysis processes. For the inverse planning optimization, a doughnut‐shaped PTV with an inner OAR was drawn and registered on the tree‐dimensional phantom computed tomography image set [Fig. 3(A)]. This planning exercise applied 9 coplanar intensity‐modulated fields. After DVO processing, the leaf motion calculation was performed using an intensity level of 20 and the multiple static segments method. Fig. 3(B) presents a schematic of the inverse planning beam arrangement.

**Figure 4:**
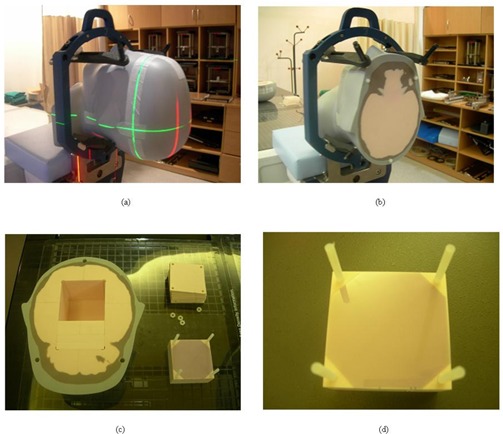
Setup for the planned irradiation: head phantom with a stereotactic head ring, and the structure of the phantom. (A) Phantom setup with head ring. (B) Truncated fixation section of the phantom. (C) Structure of the irradiated portion of the phantom. (D) Film cassette slabs (6.35 cm) containing 13 levels of films, and Gafchromic EBT film.

After the planning processes, an irradiation session was performed using a head phantom in a stereotactic head ring (BrainLAB, Feldkirchen, Germany) for stereotactic fixation (Fig. 4). In addition, a polystyrene solid phantom was used to compare the dose fluence of field 5 (post) with the measured value. The dose fluence, which was transferred from the Eclipse planning system, was imported using the OmniPro IMRT software, and the dose distribution was generated under the same geometry. The generated virtual dose distribution based on the imported fluence is known as the “desired dose distribution,” which should be close to the actual measured dose distribution. In our experiment, the desired dose distribution was compared with the measured data from the MLC files for leaf gaps of 0, 1, and 2 mm. The 2‐mm dosimetric leaf gap was taken as the desired value from the MLC‐based small‐beam dosimetry in a previous experiment. During field 5 irradiation, each planned MLC file contained 32 sequences of multiple static segments for intensity modulation.

## III. RESULTS

### A. Dosimetric static leaf gap

We noted a marked difference in profile measurements between the MLC‐based and collimated small beams. As shown in Table 1, the MLC‐based small‐beam fields were larger than the collimated fields were. The four stereotactic detectors all gave similar results. Based on the results shown in Table 2, the static dosimetric leaf gap, which was defined as Δ (radiation field–calibrated light field) here, was set to 2 mm.

**Table 1:** Radiation field widths for multileaf collimator (MLC)–based and collimated (Coll) small‐beam profile results

Detector		1×1 cm		2×2 cm		3×3 cm		4×4 cm	
		MLC	Coll	MLC	Coll	MLC	Coll	MLC	Coll
CC01	RF	1.19	0.95	2.20	2.02	3.22	3.02	4.23	4.06
	P	0.38	0.29	0.43	0.36	0.46	0.33	0.45	0.35
SFD	RF	1.23	0.95	2.19	1.94	3.25	2.98	4.21	4.01
	P	0.28	0.22	0.33	0.25	0.34	0.27	0.37	0.27
EDR2	RF	1.16	0.95	2.15	1.95	3.20	2.97	4.18	4.00
	P	0.33	0.24	0.37	0.27	0.40	0.29	0.39	0.30
EBT	RF	1.22	0.97	2.19	1.96	3.18	3.03	4.21	4.01
	P	0.39	0.22	0.46	0.23	0.47	0.25	0.47	0.28

CC01=CC01 ionization chamber (Wellhofer Dosimetrie, Louvain‐la‐Neuve, Belgium); RF=50% width at a source‐to‐axis distance of 100 cm; P=80%–20% width at a source‐to‐axis distance of 100 cm; SFD=stereotactic field detector(Wellhofer Dosimetrie); EDR2 = radiographic extended dose range film (Eastman Kodak Company, Rochester, NY); EBT = radiochromic film (International Specialty Products, Wayne, NJ).

**Table 2:** Dose–volume histograms of the three different plans for the planning target volume (PTV) and inner organ at risk (OAR), plus an analogy within a dose difference of 1%

Leaf gap	Structure	Pres dose	Treatment (%)	Volume (cm^3^)	Minimum (cGy)	Maximum (cGy)	Mean (cGy)	Median (cGy)	STD (cGy)
2 mm	Inner OAR	500.0	95.0	0.3	272.6	394.3	339.6	339.7	22.42
1 mm	Inner OAR	500.0	95.0	0.3	271.7	393.6	338.4	338.6	22.55
0 mm	Inner OAR	500.0	95.0	0.3	270.5	391.9	336.6	336.7	22.30
2 mm	PTV	500.0	95.0	6.1	447.1	549.3	530.0	537.2	18.63
1 mm	PTV	500.0	95.0	6.1	444.2	550.0	529.9	537.0	18.93
0 mm	PTV	500.0	95.0	6.1	445.5	550.9	529.9	537.0	19.21

STD=Standard deviation.

### B. Inverse planning results with various dosimetric leaf gaps

In the earlier experiment with dosimetric leaf gap using several stereotactic detectors, a 2‐mm gap was determined for the 6‐MV photon beam, and inverse planning was carried out to determine the discrepancy in the planning results that arose from the leaf‐gap settings. The final dose distributions with the three different leaf gaps were very close to each other in the planning results. The dose–volume histograms of the doughnut‐shaped PTV and inner OAR were analyzed based on the results. As shown in Table 2, the dose differences were in good agreement, within 1%.

### C. Imported dose fluence versus measurement comparison

**Figure 5:**
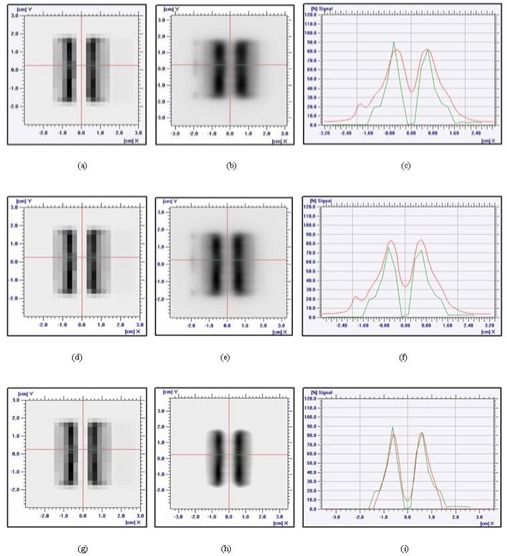
Comparison of the imported dose fluence map with an extended dose range (EDR2) film measurement of field 5. The setup geometry used a source‐to‐surface distance of 95 cm and a depth of 5 cm. (A,D,G) Dose fluence, (B,E,H) EDR2 measurement, and (C,F,I) comparison from leaf motion calculation based on leaf gaps of 0 mm, 1 mm, and 2 mm respectively. Green lines represent the data calculated by the Eclipse planning system (Varian Medical Systems, Palo Alto, CA), and red lines represent the measurements according to the static dosimetric leaf gaps.

In this experiment, field 5 (gantry angle 180 degrees, post) was used in a comparison of the imported dose fluence, which was the “desired dose distribution, with the measured value, which was the “actual dose distribution” measured using EDR2 film. The chosen profiles were offset by 2.5 mm so as to be positioned in the middle of the leaf, thus avoiding an interleaf gap and considering the round‐leaf gap effect. We compared the dose distributions measured for each of the three different gap‐generated LMCs with the dose fluence imported into the OmniPro IMRT software. The maximum dose differences for the inside PTV, outside PTV, and inner OAR were, respectively, 22.3%, 20.2%, and 35.2% for the 0‐mm leaf gap; 17.8%, 22.8%, and 30.8% for the 1‐mm leaf gap; and 5.5%, 8.5%, and 6.3% for the 2‐mm leaf gap. In the case of the 2‐mm leaf gap, the dose distributions in the PTV and inner OAR showed relatively good agreement (Fig. 5).

### D. Human head phantom study

**Figure 6:**
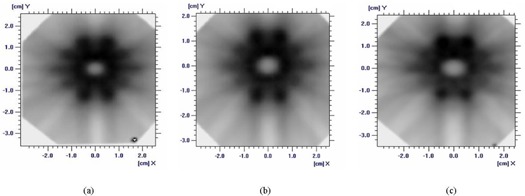
Nine‐coplanar‐field intensity‐modulated radiosurgery on the Gafchromic EBT film (6.35×6.35 cm) using a human head phantom with a stereotactic head ring, and a leaf gap of (A) 0 mm, (B) 1 mm, or (C) 2 mm.

**Figure 7:**
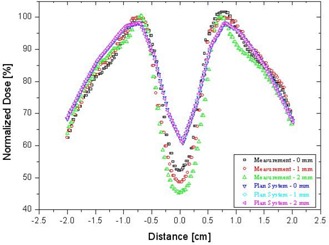
Comparison of the planned dose distributions with the measured dose distributions using three different leaf gaps (0, 1, and 2 mm).

We compared the dose distribution calculated by the Eclipse planning system with the data measured using the human head phantom (Figs. 6 and 7). The final dose calculation can contain errors if it is based on commissioning measurements that use a standard ion chamber.^(1)^ We addressed that problem by comparing the dose distribution from the Eclipse planning system with the direct measurements made using various static dosimetric leaf gaps. Fig. 7 shows that a large difference occurs between the planning and the measured dose in the PTV and OAR regions. In the gap plus OAR region, no area showed a dose value of ≤60% in the planned results, and the widths between 60% were 0.40, 0.51, and 0.63 mm for 0‐, 1‐, and 2‐mm gap measurements respectively (Table 3). The maximum dose differences in the gap measurements of the inner OAR were 16.3%, 12.9%, and 9.2% less than the planned dose from the Eclipse planning system for the 0‐, 1‐, and 2‐mm gap measurements respectively. As shown in Table 4, the planned results and the measured dose distribution showed a large discrepancy. This discrepancy is believed to be caused by the Eclipse planning system calculating the dose distribution based on the general measured beam data from the collimated beam fields of 4 – 40 cm, which means that the measured fields were not based on the MLC beam and involved relatively large field sizes.

**Table 3:** Comparison of the planned dose distribution with the measured isodose level widths for a 9‐field intensity‐modulated radiosurgery plan

		Gap+inner OAR		
		Isodose level width (mm)		
	50%	60%	70%	80%
Plan	—	—	0.44	0.73
0‐mm leaf gap	—	0.40	0.59	0.75
1‐mm leaf gap	0.18	0.51	0.69	0.84
2‐mm leaf gap	0.37	0.63	0.79	0.93

OAR=organ at risk.

**Table 4:** Dose comparison of 9‐field intensity‐modulated radiosurgery plan and the measurements by static dosimetric leaf gap

Leaf gap (mm)		Inner OAR		Gap+inner OAR		Doughnut‐shaped PTV	
		Minimum	Maximum	Minimum	Maximum	Minimum	Maximum
Plan	0, 1, 2	61.5	66.0	61.4	89.8	88.0	98.3
Measurement	0	52.3	60.4	52.3	94.2	93.9	101.7
	1	48.6	56.3	48.6	92.1	89.5	100.0
	2	45.2	50.9	45.2	88.5	83.7	100.4

OAR=organ at risk; PTV=planning target volume.

## IV. DISCUSSION

Our study shows how the leaf gap characteristics affect inverse planning results and the actual dose distribution. The beam data used in the Eclipse planning system were commissioned using a standard detector in a conventional planning system. Moreover, the measured data input into the configuration came from a focused jaw–collimated beam and not from MLC‐based fields. In the single focused MLC, such as the Varian 120 Millennium model, a potential for dosimetric errors exists because of the round leaf‐end effect and the leaf gap setting.

The present study dealt with the properties of a MLC‐based small beam as compared with the properties of a collimated beam. In addition, the planned dose distribution was compared with the delivered dose using the multiple static segments method with various leaf gaps. As seen in Table 1, clear differences in radiation field sizes were demonstrated between the collimated and the MLC‐based small beams (square of 1, 2, 3, and 4 mm). Some authors have reported that an incorrect radiation field offset, which is the difference between the radiation field and the calibrated light field at source‐to‐axis distance, would result in a dose error to the interior of a target volume.^(5)^

The present study also shows that a LMC based on an inadequate dosimetric leaf gap could be the source of dosimetric errors in the PTV and the OAR. Fig. 5 shows that the measurement result based on the MLC file with a 2‐mm leaf gap coincided well with the dose fluence from the DVO. Therefore, a well‐defined dosimetric gap can reduce dosimetric errors, particularly in delicate IMRS treatments.

As shown in Fig. 7, the final dose distributions in the PTV and inner OAR region as calculated by the Eclipse planning system for leaf gaps of 0, 1, and 2 mm were quite different from the measured values. Table 4 shows a large dose difference of 1.3% – 12.7% in the measurement with the dose distribution generated from MLC files. The planned results were similar, and measurements showed a large dose difference associated with the various leaf gaps. The reason that the results in Fig. 7 contrast with those in Fig. 5 is the different sources for the dose calculation. The desirable optimized dose fluence from dose–volume optimization of inverse planning and leaf motion calculation was based mainly on the built‐in IMRT DVO engine (Fig. 5); the dose distributions in Fig. 7 were recalculated using the general beam data kernel commissioned with a standard‐volume ionization chamber (around 0.1 cm^3^). These results strongly suggest that plans generated by a commercial inverse planning system commissioned using general collimated field data could have similar errors in the calculated dose distributions for small‐beam IMRT or IMRS.

## V. CONCLUSION

Although much discussion has surrounded small beamlet use in IMRT, small‐beam dosimetry, and stereotactic detectors, less attention has been paid to the relationship between beam data obtained during commissioning and the actual irradiation results that depend on these critical parameters. Inadequate determination of dosimetric static leaf gap during configuration of the treatment planning system can produce dosimetric error in the final dose calculation, which can sometimes be confused with unwanted quality assurance results of IMRT or IMRS. To summarize, it is worth reiterating the proposition presented earlier in this paper: optimized dose fluence showed good agreement with the optimal dosimetric leaf‐gap‐corrected measurements, but a potential for discrepancy in the calculated dose distribution commissioned by a standard‐volume ionization chamber remains. An appropriate dosimetric leaf gap setting is critical during the commissioning of an inverse planning system. An incorrect setting can produce large dose delivery errors, particularly in delicate IMRS treatments.

## ACKNOWLEDGMENTS

This work was supported by the Korea Science and Engineering Foundation (KOSEF) grant funded by the Korea government (MOST) (No. M20706000007‐07M0600‐00710)
